# *Lysimachia tianmaensis* (Primulaceae), a new species from Anhui, China

**DOI:** 10.3897/phytokeys.98.23751

**Published:** 2018-05-16

**Authors:** Ying Wang, Wen Ma, Shoubiao Zhou, Kun Liu

**Affiliations:** 1 Anhui Provincial Key Laboratory of the Conservation and Exploitation of Biological Resources, College of Life Sciences, Anhui Normal University, Wuhu 241000, China; 2 Anhui Provincial Engineering Laboratory of Water and Soil Pollution Control and Remediation, College of Environmental Science and Engineering, Anhui Normal University, Wuhu 241002, China; 3 College of Life Sciences, Shaanxi Normal University, Xi’an 710119, China

**Keywords:** *Lysimachia*, species nova, China, taxonomy

## Abstract

A new species of *Lysimachia* (Primulaceae), *Lysimachia tianmaensis* K. Liu, S.B. Zhou & Ying Wang **sp. nov.**, is described and illustrated from Jinzhai County, Anhui, China. It is endemic to Dabieshan Mountain, China. The new species has yellow flowers and belongs to the subgenus Lysimachia section Nummularia series *Grammicae*. It is very easily distinguishable from other related species by having alternate leaves with brown patches beneath and an auriculated leaf base.

## Introduction

*Lysimachia* Linnaeus is one of the largest genera of Primulaceae s. l. and it comprises about 200 species, mainly distributed in the temperate and subtropical parts of the northern hemisphere, as well as in some tropical mountain regions (Chen and Hu 1979, Hu and Kelso 1996, Marr and Bohm 1997, Hao et al. 2004, Julius et al. 2016). On the whole, it is almost cosmopolitan, but the greatest concentration of the species occurs in China (with ca. 140 species; Chen et al. 1989, Hu and Kelso 1996). Some new species in *Lysimachia* are still being found (Peng and Hu 1999, Shao et al. 2004, Shao et al. 2006, Zhang et al. 2006, Yan and Hao 2012, Liu et al. 2014a, 2014b, Estes et al. 2015, Zhou et al. 2015, Baskose et al. 2016, Julius et al. 2016).

In 2007, during the course of checking specimens in the herbaria of Anhui Normal University, a specimen of *Lysimachia* caught the authors’ attention. This plant was collected by Shen in 1983 from Jinzhai County, Anhui Province and was not identified. This plant has alternate leaves and an obvious broadly-winged petiole with an auriculate base. It should thus represent an undescribed species, as this character combination is not known from any other species. In 2008–2009, the authors made several botanical expeditions to Tianma Nature Reserve, in Jinzhai County, Anhui Province. Many populations of this plant were found bearing flowers or fruits there. In this paper, this plant and related species were comparatively studied.

## Materials and methods

Vouchers of *Lysimachia tianmaensis* were collected from Tianma National Nature Reserve of Anhui. Gross morphology and phenology data were obtained during the field expedition. Descriptions were collected from living plants.

## Taxonomy

### Lysimachia tianmaensis

Taxon classificationPlantaeORDOFAMILIA

K. Liu, S.B. Zhou & Ying Wang sp. nov.

urn:lsid:ipni.org:names:77178766-1

Figures 1 , 2 , 3

#### Type.

CHINA. Anhui Province: Jinzhai County, Tianma National Nature Reserve, growing at margins of mountain roads, elevation ca. 1165 m, 1 June 2009 (fl.), Kun Liu 2009042 (holotype: ANUB!; isotypes: ANUB!, IBK!).

#### Diagnosis.

*Lysimachia tianmaensis* is similar to *Lysimachia grammica* Hance in the alternate leaves, but differs by having a larger blade with brown patches beneath, an auriculate leaf base and subcapitate inflorescences.

#### Description.

Herbs perennial, 15–45 cm tall. Stems often many, erect or arcuate at base, terete, simple or short branched, with tangled multicellular hairs. Leaves alternate, occasionally opposite on lower part; petiole 3–25 mm, broadly winged, base auriculate on leaves from middle and lower part of stems and branches. Leaf blades ovate to ovate-elliptic, rarely ovate-lanceolate, 1.5–5.5 × 1.0–3.5 cm, abaxially multicellular hairs, brown patches, adaxially pubescent, base broadly cuneate to subrounded, apex acute to subobtuse; veins 2 or 3 pairs, inconspicuous. Flowers solitary, in axils of apically diminished leaves, often in shortened, nearly capitate inflorescences at apex of stems and branches. Pedicel densely covered with multicellular hairs; lowest pedicels 2–3 cm, gradually reduced in length in upper flowers, recurved in fruit. Calyx lobes ovate-lanceolate, 6–7 × 1–1.4 mm, abaxially sparsely pubescent. Corolla yellow; tube 0.5–1 mm; lobes ovate or rhomboid-ovate, 8–11 × 5–7.5 mm, transparent glandular. Filaments connate basally into a 0.5–1 mm high ring, free parts 2.5–3 mm; anthers dorsifixed, opening by lateral slits. Ovary pubescent; style 5–6 mm. Capsule subglobose, 3.5–5 mm in diam. Fl. Apr–Jun.

**Figure 1.**
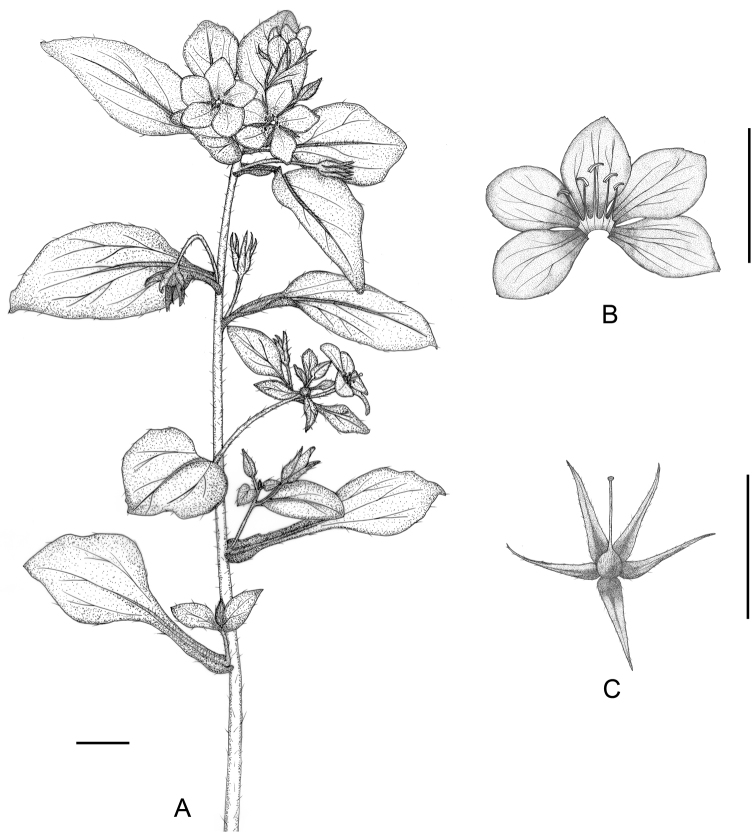
*Lysimachia tianmaensis* sp. nov. (**A**) the upper part in flowering period **B** opened corolla showing stamens **C** pistil and calyx. Scale bars = 1 cm.

**Figure 2.**
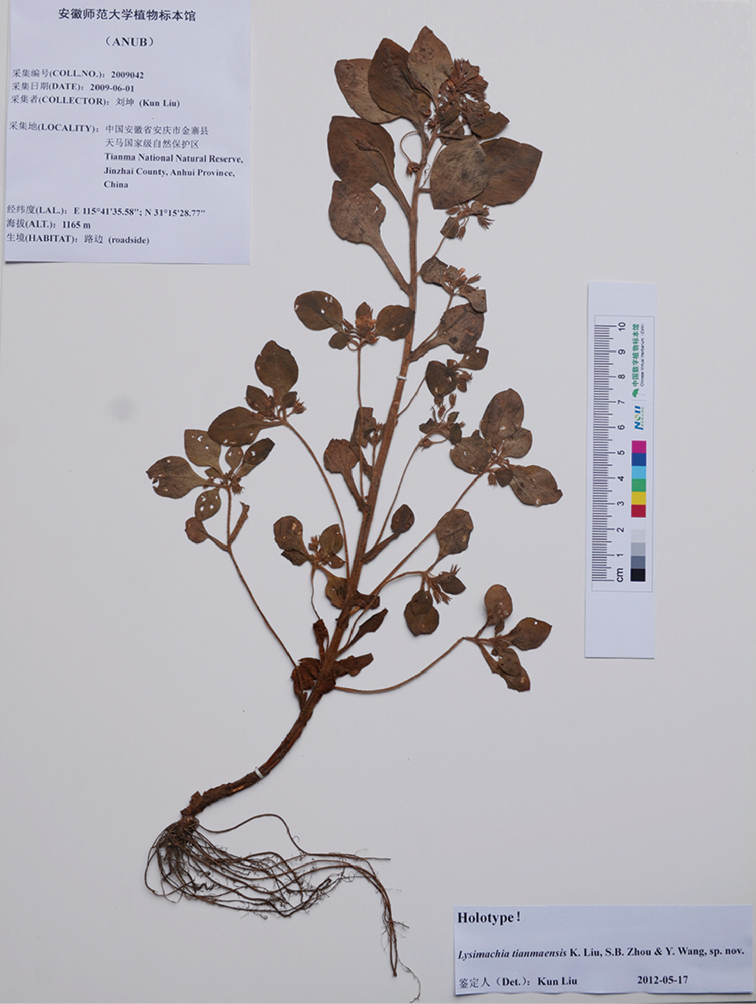
Holotype sheet of *Lysimachia tianmaensis* sp. nov.

**Figure 3.**
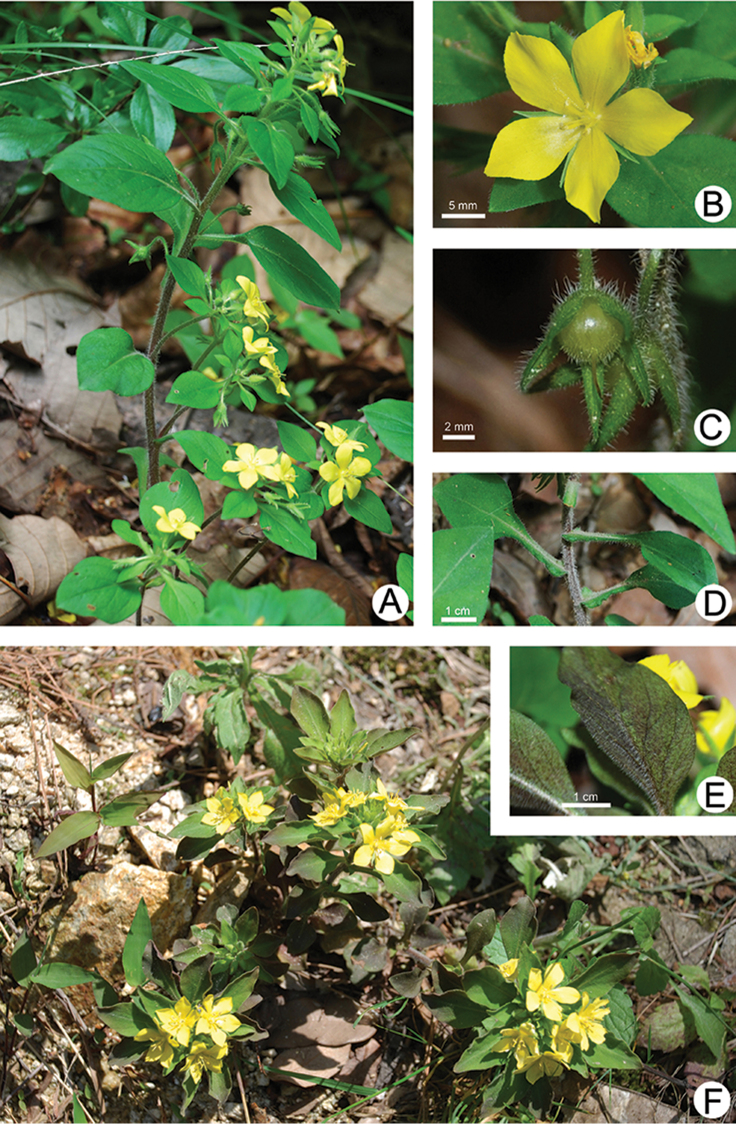
*Lysimachia tianmaensis* sp. nov. **A** plant in flowering **B** flower **C** young fruit **D** leaves showing winged petiole with auriculate base **E** blades showing brown patches abaxially **F** habit in flowering.

#### Additional collection.

CHINA. Anhui Province: Jinzhai County, Tiantangzhai, ca. 650 m, 17 June 2008, *K. Liu 2008056* (ANUB); Jinzhai County, Mazongling mountain, 950 m, 1 June 2009, *K. Liu 2009038* (ANUB); Jinzhai County, Tiantangzhai, ca. 700 m, 1983, *X.S. Sheng 1437* (ANUB); Jinzhai County, Baimazhai, 900 m, 18 May 1984, *G. Yao 9004* (NAS); Jinzhai County, Baimazhai, 700 m, 23 May 1984, *G. Yao 9056* (NAS); Jinzhai County, Gubeizhen, 720 m, 4 May 2016, *J.W. Shao ANUB00569* (ANUB).

#### Distribution and habitat.

*Lysimachia tianmaensis* is endemic to Dabieshan Mt., China (including Jinzhai County, Yingshan County etc.), growing at margins of mountain woodlands, roadsides or under broad-leaved forests at altitudes of 600–1200 m.

#### Etymology.

The epithet “tianmaensis” is derived from the type locality, Tianma National Nature Reserve, Jinzhai Xian, Anhui Province, China.

#### Vernacular name.

China: tian ma guo lu huang.

#### Phenology.

Flowering April–June, fruiting June–August.

#### Conservation status.

A large number of populations of *Lysimachia tianmaensis* were found during the extensive investigation in Tianma National Nature Reserve. This species is also distributed in other areas in Dabieshan Mt. as well as Tianma National Nature Reserve. This species often grows under broad-leaved forests above 600 m. This species is fairly common there and therefore proposed as Least Concern following the IUCN Red List Criteria (IUCN 2016).

## Discussion

*Lysimachia tianmaensis* is quite distinct from all other species in subgenus Lysimachia. Its morphological affinity is with *L. grammica*, *L. remota* Petitmengin and *L. pseudohenryi* Pampanini, but it can be easily distinguished by some characters (Table 1). *L. remota*, a member of subgenus Lysimachia, section Nummularia, series *Deltoideae*, is characterised by opposite leaves with sparsely transparent glandular punctate. *L. pseudohenryi* has opposite leaves and terminal racemes, often nearly capitate, belonging to subgenus Lysimachia, section Nummularia, series *Phyllocephalae*. Due to the opposite leaves in both *L. remota* and *L. pseudohenryi*, *L. tianmaensis* can be distinguished from them by its alternate leaves with brown patches beneath and auriculate leaf base. Taking into consideration the existence of alternate leaves and the filaments connate into a ring at base, *L. tianmaensis* should be a member of the subgenus Lysimachia, section Nummularia, series *Grammicae*, according to the classification system of the genus modified by Chen and Hu (1979). Series *Grammicae* is a well-defined group, so far consisting of only one species. *L. grammica* is a widely distributed species with its distribution centre in Anhui, Henan, Hubei, Jiangsu, Jiangxi, S Shaanxi and Zhengjiang and the new species is endemic to Dabieshan Mt. However, the new endemic species rarely, if ever, co-occurs with the widespread *L. grammica* in intermixed populations because of the distinct altitudes for each natural habitat (*L. tianmaensis*: 600–1200 m; *L. grammica* 0–600 m, rarely to 800 m). The new species has a larger lamina with brown patches beneath than that of *L. grammica* with blank glandular striates. Moreover, the leaves of the new species are characterised with an obvious auriculate base. Based on these characters, *L. tianmaensis* can be very readily distinguished from *L. grammica*.

**Table 1.** Diagnostic character differences amongst *Lysimachia tianmaensis*, *L. grammica*, *L. remota* and *L. pseudohenryi*.

Species	*L. tianmaensis*	*L. grammica*	*L. remota*	*L. pseudohenryi*
Source	This study	Hu and Kelso (1996)	Hu and Kelso (1996)	Hu and Kelso (1996)
Leaf	alternate, occasionally opposite on lower part; abaxially brown glandular punctate; base auriculate on middle and lower part of stems and branches	opposite on lower part, alternate on upper part; black glandular stripes	opposite, occasionally alternate on upper part; sparsely transparent glandular punctate	opposite; sparsely transparent glandular
Blade size (cm)	1.5–5.5 × 1.0–3.5	1.3–3.5 × 0.8–2.5	1.5–3.2 × 0.7–2.0	2–8 × 0.8–2.5
Infloresecence	flowers solitary, in axils of apically diminished leaves, often abbreviated, nearly capitate at apex of stems and branches	flowers solitary, in axils of upper leaves	flowers solitary, in axils of upper leaves, or capitate with flowers aggregated near apex of stems	racemes terminal, abbreviated, often nearly capitate
Filament	filaments connate basally into a 0.5–1.0 mm high ring	filaments connate basally into a ca. 0.5 mm high ring	filaments connate basally into a 0.5–1.0 mm high ring	filaments connate basally into a 2–3 mm high tube
Corolla	transparent glandular	brown glandular stripes	transparent glandular	transparent glandular

## Supplementary Material

XML Treatment for Lysimachia tianmaensis

## References

[B1] Baskose I Keskin A Gurbanov K (2016) *Lysimachia savranii* (Primulaceae), a new species from the eastern Taurus in Turkey. Phytotaxa 267(3): 228–232. https://doi.org/10.11646/phytotaxa.267.3.6

[B2] Chen FH Hu CM (1979) Taxonomic and phytogeographic studies on Chinese species of *Lysimachia*. Zhiwu Fenlei Xuebao 17: 21–53.

[B3] Chen FH Hu CM Fang YI Cheng CZ (1989) Primulaceae. In: Chen FH Hu CM (Eds) Flora Reipublicae Popularis Sinicae vol. 59. Science Press, Beijing, 3–133.

[B4] Estes D Shaw J Mausert-Mooney C (2015) *Lysimachia lewisii* (Primulaceae): a new species from Tennessee and Alabama. Phytoneuron 17: 1–15.

[B5] Hao G Yuan YM Hu CM Ge XJ Zhao NX (2004) Molecular phylogeny of *Lysimachia* (Myrsinaceae) based on chloroplast trnL-F and nuclear ribosomal ITS sequences. Molecular Phylogenetics and Evolution 31(1): 323–339. https://doi.org/10.1016/S1055-7903(03)00286-0 15019628 10.1016/S1055-7903(03)00286-0

[B6] Hu CM Kelso S (1996) Primulaceae. In: Wu ZY Raven PH (Eds) Flora of China, vol. 15. Science Press, Beijing, and Missouri Botanical Garden Press, St Louis, Missouri, 39–189.

[B7] Julius A Tagane S Naiki A Gutierrez-Ortega JS Suddee S Rueangruea S Yahara T Utteridge T (2016) *Lysimachia kraduengensis* (Primulaceae), a new species from northeastern Thailand. Phytotaxa 289(1): 69–76. https://doi.org/10.11646/phytotaxa.289.1.5

[B8] Liu K Hong X Zhou SB Chen YS Tang CF Xu HJ (2014a) A new species of *Lysimachia* (Myrsinaceae) from Dabieshan Mountain, China. Plant Systematics and Evolution 300(7): 1615–1620. https://doi.org/10.1007/s00606-014-0986-z

[B9] Liu K Zhou SB Chen YS Hong X (2014b) *Lysimachia dabieshanensis* sp. nov. (*Primulaceae*), a new species from Dabieshan Mountain, China. Phytotaxa 174(2): 119–122. https://doi.org/10.11646/phytotaxa.174.2.8

[B10] Marr KL Bohm BA (1997) A taxonomic revision of the endemic Hawaiian *Lysimachia* (Primulaceae) including three new species. Pacific Science 51: 254–287.

[B11] Peng CI Hu CM (1999) *Lysimachia chingshuiensis* (Primulaceae), a new species from eastern Taiwan. Bulletin Academia Sinica Taipei 40: 49–52.

[B12] Shao JW Zhang XP Guo XH (2004) A new species of *Lysimachia* in Primulaceae. Bulletin of Botanical Research 24: 389–391.

[B13] Shao JW Zhang XP Guo XH (2006) *Lysimachia dextrorsiflora* X. P. Zhang, X. H. Guo & J. W. Shao, a new species of Primulaceae from China. Zhiwu Fenlei Xuebao 44(5): 589–594. https://doi.org/10.1360/aps050135

[B14] Yan HF Hao G (2012) *Lysimachia huchimingii* sp. nov. (Primulaceae) from China. Nordic Journal of Botany 30(4): 443–445. https://doi.org/10.1111/j.1756-1051.2012.01401.x

[B15] Zhang MD Shui YM Chen WH Wei ZD (2006) *Lysimachia gesneroides* (Myrsinaceae), a new species from Yunan and Vietnam. Annales Botanici Fennici 43: 317–319.

[B16] Zhou JJ Yu XL Deng YF Yan HF Lin ZL (2015) *Lysimachia huangsangensis* (Primulaceae), a new species from Hunan, China. PLoS One 10(7): e0132713. https://doi.org/10.1371/journal.pone.0132713 10.1371/journal.pone.0132713 PMC4511667 26201028

